# Enhancement of hydrophobicity and UV aging resistance of Poly (p-phenylene benzobisoxazole) fibers modified by fluorosilane and UV absorber

**DOI:** 10.1038/s41598-019-45199-8

**Published:** 2019-06-13

**Authors:** Zhihua Li, Yu Yang, Hui Li, Lanlan Liu, Dehua Zou

**Affiliations:** 1 College of Material Science and Engineering, Central South University, Changsha, 410000 China; 2 Key Laboratory of Nonferrous Metal Materials Science and Engineering Ministry of Education, Central South University, Changsha, 410000 PR China; 3 State Grid Human Maintenance Company, Changsha, 410000 PR China; 4 Hunan Province Key Laboratory of Intelligent Live Technology and Equipment (robot), Changsha, 410000 PR China

**Keywords:** Materials science, Engineering

## Abstract

Poly (p-phenylene benzobisoxazole) (PBO) fibers were functionalized by 3-Glycidoxypropyltrimethoxysilane (KH-560) and then coated with 1 H, 1 H, 2 H, 2H-Perfluorodecyltrimethoxysilane (HFTES) and UV absorber (UV-328) to improve hydrophobicity and UV aging resistance. The chemical compositions of PBO fibers before and after modification were analyzed by XPS and surface morphologies were observed by SEM. The hydrophobicity of PBO fibers was evaluated by the measurement of contact angle for water and the UV aging resistance was evaluated by the tensile strength retention ratio of PBO fibers. The results showed that KH-560 was successfully introduced onto the PBO fiber surface. The contact angle for water was increased by 128% from 51.7° to 127°, suggesting a huge improvement of hydrophobicity. The UV aging resistance of PBO fibers was also greatly improved. After 400 h UV exposure, the tensile strength retention ratio was increased to 53%, which was much higher than that of 21.4% for pristine PBO fibers. And the results of variable coating times demonstrated that the optimal UV aging resistance was obtained by coating three times.

## Introduction

With the increase of electric power consumption, ultra-high voltage AC transmission at 1000 kV AC and above voltages have attracted wide attention to achieve long distance and bulk capacity power transmission. Thereupon, live working plays a crucial role in ensuring the safety and stability of equipment and power system. While traditional live working tools made of titanium alloy are too bulky and heavy to manipulate, there is an urging need to find new material to make them simple and flexible. Over the past few decades, the field of high performance fiber has witnessed considerable growth. As a representative of high-performance fibers, Poly (p-phenylene benzobisoxazole) (PBO) is known for its excellent properties, such as outstanding mechanical properties, good thermal stability and chemical resistance^1–3^. Based on these superior characteristics, PBO fibers are extensively applied in the field of military, aerospace and general industry^4–6^. Especially, the superior strength of PBO fiber (tensile strength of 5.8 Gpa and tensile modulus of 180 Gpa) with low density of 1.54 g/cm^3^ also makes it an ideal candidate for application in live working.

In order to practical use in harsh environmental conditions, there still remain some challenges to overcome. Due to the lack of chemical active groups, PBO fiber has a low surface energy of 32.2 mJ/m^2^ and a contact angle for water of 51.7°, which is insufficient to meet the requirements of insulation for live work tools. At the same time, in-service studies have revealed that the strength of PBO fibers significantly reduce in the mild environment under UV exposure^7–9^. And water molecules can penetrate through defects on the PBO surface, which results in more internal defects and further degradation of PBO fiber^10,11^. Unfortunately, there are few studies related to improve the hydrophobicity and UV aging resistance of PBO fiber simultaneously.

Given the chemical inert and poor interfacial adhesion with resin, it’s necessary to functionalize the surface of PBO fiber. Among various surface modification techniques, such as plasma treatment^12,13^, acid treatment^14,15^, coupling agent treatment^16,17^ and direct fluorination^18,19^, coupling agent treatment is a simple and facile approach with less damage to PBO fiber. In our present work, for the improvement of hydrophobicity and UV aging resistance, the PBO fiber was firstly treated by coupling agent of 3-Glycidoxypropyltrimethoxysilane (KH-560) and then coated by a silicone modified epoxy resin coating containing 1H, 1H, 2H, 2H-Perfluorodecyltrimethoxysilane (HFTES) and UV absorber (UV-328). X-ray photoelectron spectroscopy (XPS) and scanning electron microscope (SEM) were performed to characterize the surface morphology and structure, angle contact measurement and mechanical analysis were used to investigate the hydrophobicity and ultraviolet stability of modified PBO fiber. Meantime, the effect of coating times was also investigated. The results indicate that a hydrophobic and UV resistant PBO fiber could be prepared using the facile and effective method proposed herein. This work will pave the way for the further research of the applications of PBO fiber in live working.

## Results and Discussions

**Figure 1:**
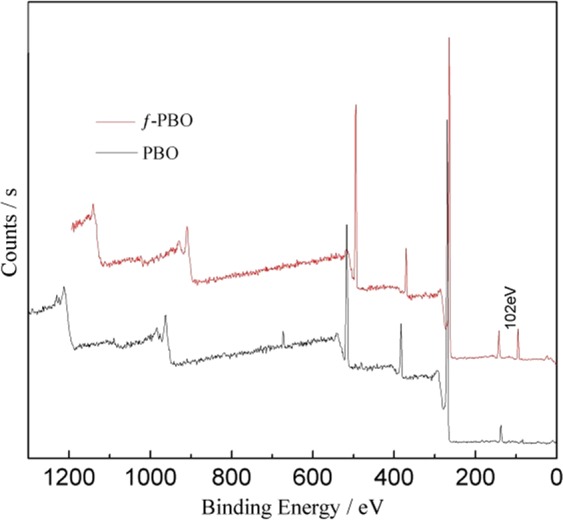
XPS spectra of PBO fibers before and after functionalization.

Due to the smooth and chemical inert surface of PBO fiber, there is poor interfacial adhesion between PBO fiber and resin matrix. Therefore, the surface modification of PBO fiber is necessary. The surface chemical compositions of pristine PBO fibers and ƒ-PBO fibers are assessed by XPS as shown in Fig. 1. From the wide-scan survey XPS spectra, both the peaks of carbon, oxygen and nitrogen atoms can be found in pristine PBO fibers and ƒ-PBO fibers. After functionalized with KH-560, a new peak at 102 eV emerges in ƒ-PBO fibers corresponding to the Si2p^20^, which suggests that KH-560 is successfully introduced onto the PBO fiber surface by forming covalent bond. It’s obvious that functionalization process causes a significant change on surface elemental composition as listed in Table 1. Compared to pristine PBO fibers, the proportion of oxygen and nitrogen decreases from 11% to 9% and 10% to 5%, respectively. Meanwhile, the atomic percent of Si increases from 0% to 5%, which also indicates a successful functionalization procedure.

**Table 1:** Chemical surface composition of pristine PBO and ƒ-PBO.

Samples	Atomic percent (%)			
	C	N	O	Si
Pristine PBO	79	10	11	0
ƒ-PBO	81	5	9	5

**Figure 2:**
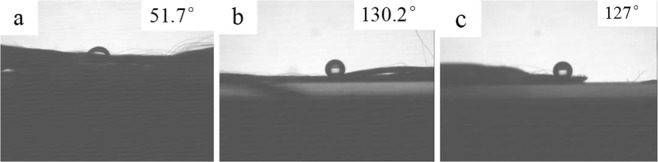
Images of a sessile drop measurement of the water contact angle on pristine PBO (**a**), HFTES/ƒ-PBO (**b**) and HFTES/UV-328/ƒ-PBO (**c**).

Contact angle is used to analyze the surface wettability of pristine PBO (a), HFTES/ƒ-PBO (b) and HFTES/UV-328/ƒ-PBO (c), as depicted in Fig. 2. In this figure, we can know that the pristine PBO fibers without chemical modification have a contact angle for water of 51.7°, which is consistent with the result of Jiang *et al*.^21^. After functionalized with HFTES and silicone modified epoxy resin, the water contact angle on fiber surface is increased by 128% from 51.7°to 130.2°, which shows a huge degrade of the wettability. Because the activated PBO fiber surface contains a large number of epoxy groups and siloxane groups, both of which can bond with the methoxy groups in HFTES and form HFTES coating on the surface of PBO fiber. Meanwhile, the methoxy group in HFTES is bonded with PBO fiber by chemical bond so that fluorine atoms are arranged on the fiber surface regularly, which can endow the fiber surface with excellent hydrophobic properties. Due to the strong surface adhesion and excellent hydrophobicity of the silicone modified epoxy resin, the hydrophobicity of the fibers is further improved. The effect of the introduction of UV-328 is also considered here. Compared to HFTES/ƒ-PBO fibers, HFTES/UV-328/ƒ-PBO fibers possess a contact angle for water of 127°. This result proves that UV-328 has no obvious effect on the hydrophobicity of the fibers.

**Figure 3:**
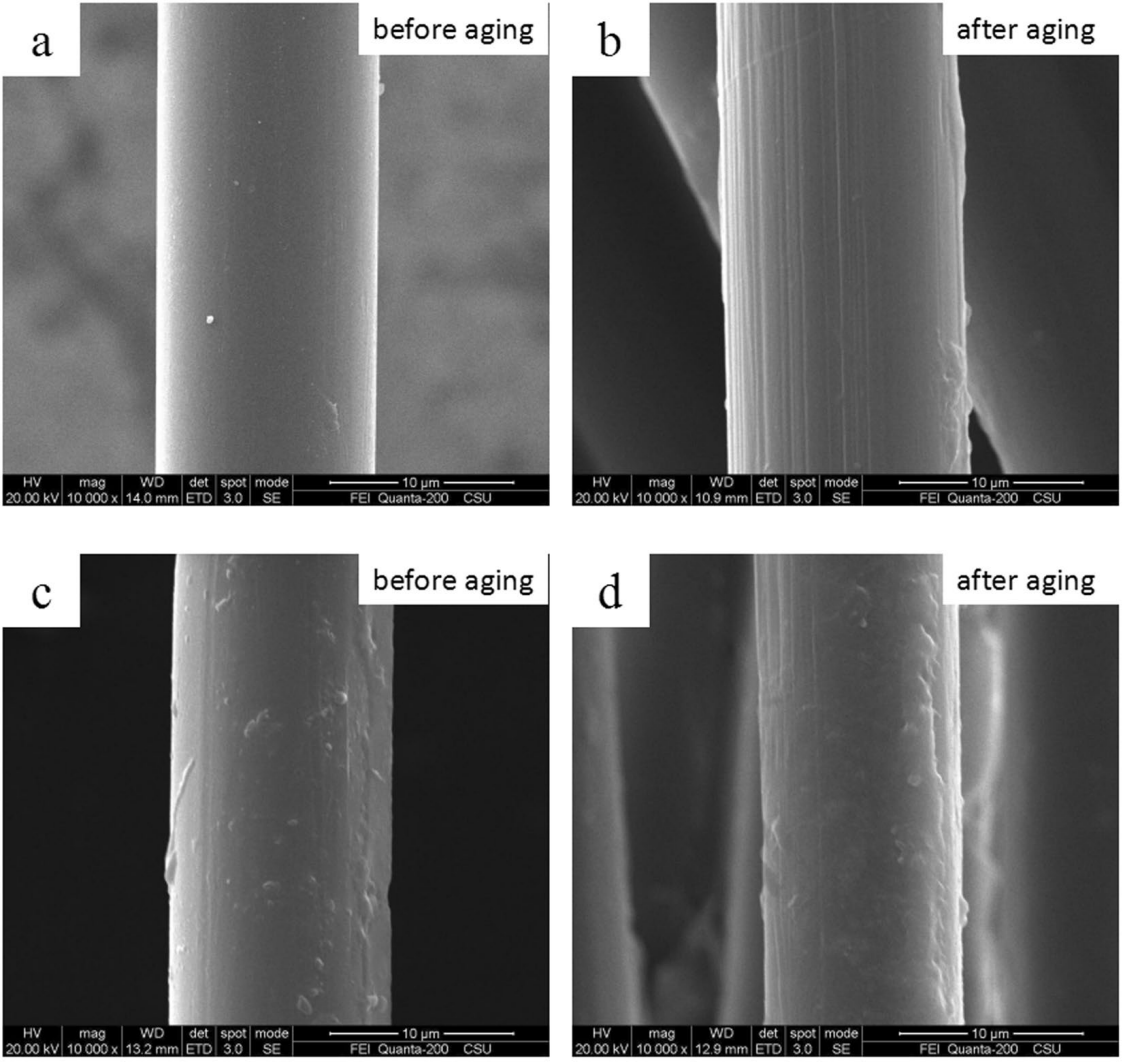
SEM images before and after UV exposure of pristine PBO (**a**,**b**) and HFTES/UV-328/ƒ-PBO (**c**,**d**).

The SEM images of pristine PBO fibers and HFTES/UV-328/ƒ-PBO fibers before and after UV aging are given in Fig. 3. For pristine PBO fiber (Fig. 3a), it is evident that the surface is quite neat and smooth before UV aging. In the case of HFTES/UV-328/ƒ-PBO fibers (Fig. 3c), the surface becomes rough covered with a film, ascribed to the deposition of HFTES and UV-328.After 400 h UV exposure, there are more grooves distribute along the axial direction on the surface of the pristine PBO fibers than those on HFTES/UV-328/ƒ-PBO fibers from Fig. 3(b,d), indicating the pristine PBO fibers suffers more severe erosion.

**Figure 4:**
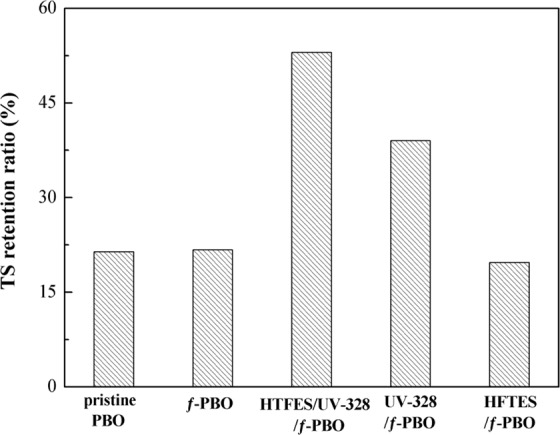
Tensile strength retentions of the PBO fibers with different coating after 400 h UV exposure.

**Figure 5:**
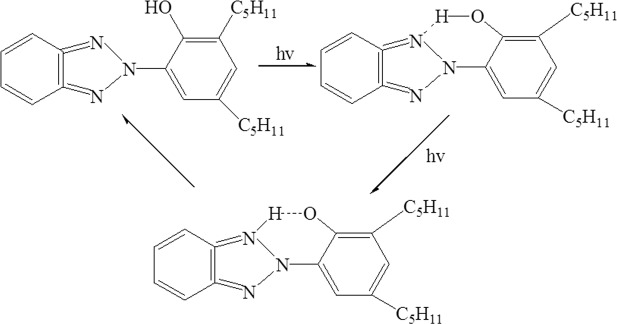
Schematic diagram of UV absorption process of UV-328.

PBO fibers have a core-shell structure, which has proved by previous work^21^. And the shell region plays an important role in undergoing external load, so the damage of a small proportion of shell region may lead to a large degrade in mechanical properties. Here the tensile strength retention of the PBO fibers is used to assess the UV resistance. As shown in Fig. 4, the TS retention ratios after 400 h UV accelerated aging of pristine PBO, ƒ-PBO, HFTES/UV-328/ƒ-PBO, HFTES/ƒ-PBO and UV-328/ƒ-PBO are 21.4%, 21.7%, 53%, 39% and 19.7%, respectively. Clearly, the TS retention ratio of UV-328/ƒ-PBO is much higher than that of pristine PBO, ƒ-PBO and HFTES/ƒ-PBO, attributed to the introduction of UV-328. The process of absorbing ultraviolet radiation for UV-328/ƒ-PBO is schematically presented in Fig. 5. The absorption of UV light by UV-328 shifts the electron cloud density from the oxygen atom to the triazole ring nitrogen atom and protons rapidly transfer to form tautomers. This tautomeric structure is very unstable, converting light energy into heat energy and returning to a more stable ground state. By contrast, HFTES/UV-328/ƒ-PBO has a highest TS retention ratio of 53%, which gives rise to a 35.9% improvement compared to UV-328/ƒ-PBO. It is mainly attributed to increase hydrophobicity of PBO fibers, which prevents water penetrating through surface defects to further deteriorate the strength of PBO fibers. Combining with the results of contact angle, it is obvious that the enhancement of hydrophobicity and UV aging resistance simultaneously for PBO fibers is effectively achieved, which is few reported in other studies of PBO fibers.

**Figure 6:**
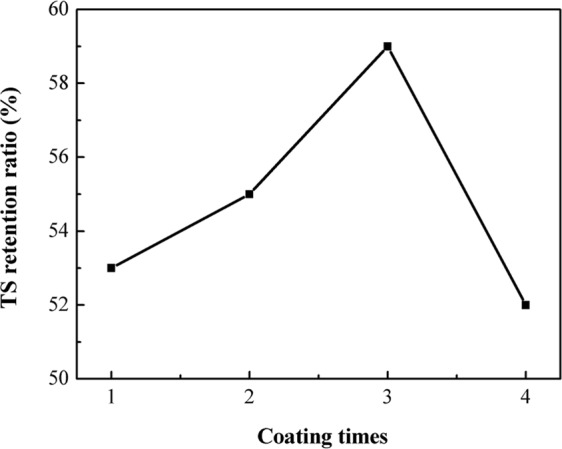
Effect of coating times on UV resistance of PBO fibers.

The effect of coating times on UV resistance of PBO fibers is also investigated (Fig. 6). As shown, the TS retention ratios of HFTES/UV-328/ƒ-PBO fibers are firstly increased and then decreased with the coating times. And a highest TS retention ratio of 59% is obtained for HFTES/UV-328/ƒ-PBO coated with three times. The key point is to achieve homogeneous distribution of UV-328 on the surface. The regions which are not covered with UV-328 evenly are susceptible to UV irritation, which can easily lead to a significantly decrease of the overall mechanical property of PBO fibers.

## Conclusion

In summary, HFTES/UV-328/ƒ-PBO fiber, a high hydrophobic and UV aging resistant PBO fiber was prepared by modification of KH-560 and coating with HFTES and UV-328. The changes of surface chemical composition for pristine PBO fibers and ƒ-PBO fibers indicate that KH-560 is successfully introduced onto the PBO fiber surface by forming covalent bond. Compared to the pristine PBO fibers, the hydrophobicity and UV resistance of the PBO treated by the coating are significantly improved. The contact angle for water is increased by 128% from 51.7°to 127°and the introduction of UV-328 has no obvious effect on the hydrophobicity of the fibers. After 400 h UV aging, the TS retention ratio of 53% for HFTES/UV-328/ƒ-PBO fibers is superior to that of 21.4% for pristine fibers. Meanwhile, the effect of coating times on UV resistance of PBO fibers is also investigated. And when coated with three times, an optimal TS retention ratio of 59% can be obtained for HFTES/UV-328/ƒ-PBO fibers. This simple and effective method may offer a novel way for PBO fibers to improve hydrophobicity and UV aging resistance simultaneously, which shows great promise for applications of live working tools.

## Method

### Materials

Poly (p-phenylene benzobisoxazole) (PBO) fibers were purchased from Toyobo Ltd., Japan. Silicone modified epoxy resin with an epoxy value of 0.08 was obtained from Wujiang Heli Resin Co. Ltd., China. Polyamide 650 with an amine value of 200 ± 20 KOH/g was supplied by Yichun Yuanda Chemical Co. Ltd., China. KH-560 and UV-328 were provided by Shanghai Yi Ji Industrial Co., Ltd., China. 1H, 1H, 2H, 2H-Perfluorodecyltrimethoxysilane (HFTES) was purchased from Mclean Reagent Co., Ltd., China. Ethyl acetate and ethanol were supplied by Sinopharm Chemical Reagent Co., Ltd., China.

### Surface functionalization of PBO

Prior to use, PBO fibers were soaked in ethanol for 24 h to remove the sizing agent and contaminants from the surface, followed by washed with distill water and dried in the oven at 120 °C for 1 h. The pretreated PBO fibers were then immersed into 5% wt KH-560/ethanol solution by ultrasonic for 10 minutes at room temperature, followed by washed with distilled water and dried at 120 °C for 1 h to obtain KH-560 functionalized PBO (ƒ -PBO) fibers.

### Preparation of high-hydrophobic and UV-resistant PBO

3 g of silicone modified epoxy resin and 0.45 g of polyamide 650 were dissolved in 15 g of ethyl acetate to obtain a solution. Then 0.09 g of HFTES and 0.5 g of UV-328 were added to the solution by ultrasonic until they mixed well. The ƒ-PBO fibers were then immersed into the above mixture at 50 °C for 10 min, followed by curing at 65 °C for 3 h, finally to obtain HFTES/UV-328/ƒ-PBO fibers. With the same method, the samples named as HFTES/ƒ-PBO fibers and UV-328/ƒ-PBO fibers were prepared without UV-328 or HFTES, respectively.

### UV accelerated aging

The samples were subject to UV irradiation (340 nm) using QUV/spray (Q-Lab, America) at 50 °C for 400 h to accelerate the UV aging process of the fibers.

### Characterization

X-ray photoelectron spectroscopy (XPS) analyses of the elemental chemical composition of PBO fibers before and after functionalization process were carried out by ESCALAB250XI XPS (ThermoFisher Scientific, America). Scanning electron microscope (SEM) morphologies of the samples were analyzed by SIRION2000 (FEI, Holland). The samples were sputtered with Au before observation.

To analyze the hydrophobicity of the samples surface, the measurement of contact angle for water was performed on DSA100 (KRUSS, German). The contact angle was calculated by following equation:

$$\theta =2\arctan \frac{2h}{d}$$

where θ is the contact angle for water; h is the height of the drop crown; D is the diameter of the bottom circle of the drop crown.

Monofilament tensile strength of the samples before and after UV aging was measured by MTS-810 electronic universal material testing machine (MTS Systems Co, China) following ASTM D 3379-15 at room temperature and 30 specimens were tested to obtain the average.
